# Raman and IR spectroscopy as a promising approach to rapid and non-destructive monitoring of chlorogenic acid in protein matrices

**DOI:** 10.3389/fchem.2025.1543663

**Published:** 2025-03-05

**Authors:** Yulia Vershinina, Elizaveta Reshetnikova, Shixian Lv, Irina Kolesnik, Olesya Kapitanova, Irina Veselova

**Affiliations:** ^1^ Chemistry Department, Lomonosov Moscow State University, Moscow, Russia; ^2^ School of Materials Science and Engineering, Peking University, Beijing, China

**Keywords:** sunflower meal, chlorogenic acid, vibrational spectroscopy, Raman, FTIR

## Abstract

Sunflower meal, a by-product of the oil extraction process from sunflower seeds, has high-quality protein content. Its low production cost, near-absence of toxic substances, and close to “ideal” amino acid composition give it several advantages over other plant-based protein sources. However, at the moment, the use of sunflower meal in the food industry is still limited. This is due to the high content of phenolic compounds (1-4 wt%), particularly chlorogenic acid. The oxidation products of these compounds easily bind to protein molecules, giving the final product a dark green color and bitter taste. Currently, there is a high demand for the development of methods for rapid monitoring of the content of phenolic compounds in plant materials without special processing at various stages of the technological process without preliminary sample preparation for analysis. In this study, we used non-destructive vibrational spectroscopy techniques–Raman and FTIR–to monitor the content of chlorogenic acid in the raw material. As a result, an approach for the determination of chlorogenic acid in sunflower meal using IR spectroscopy with limit of detection (LOD) 0.75 wt% has been proposed. Its content in the studied sample of sunflower meal was 5.6 wt%, which was confirmed by UV-spectroscopy and HPLC. The paper demonstrates the principal possibility of analyzing protein isolates using Raman scattering, with the LOD for chlorogenic acid content at 1 wt%.

## 1 Introduction

Recently, plant-based proteins have been used increasingly as an economical and versatile alternative to animal-derived proteins in human nutrition, and as functional ingredients in product formulations (Sá et al., 2020). Using oil industry waste as an alternative source of animal protein could meet protein demand worldwide (Comunian et al., 2021). One of the promising sources of vegetable protein is a by-product of sunflower processing–sunflower meal, which contains protein, fiber, minerals, and polyphenols. The valuable properties of sunflower meal include its low production costs and the almost complete absence of toxic and anti-nutritional substances, as well as allerge.

Although sunflower meal has a high protein content (between 27 wt% and 63 wt%, depending on the processing method), it is primarily used as a protein supplement for livestock and poultry. Currently, sunflower meal is under-utilized as an ingredient in food (González-Pérez et al., 2002) due to the high content of phenolic compounds (1.4–5.8 wt%) (Shiroma-Kian et al., 2008). These phenols are represented by chlorogenic acid (more than 70 wt% of all phenolic compounds), which is a complex ester of caffeic acid and quinic acid (Wildermuth et al., 2016). Under high pH (>9 and heating (>40°C), chlorogenic acid is easily oxidized and irreversibly binds to protein molecules, giving the final protein product a green color (Quan et al., 2019). Therefore, it is necessary to control the content of chlorogenic acid in sunflower meal to obtain white to cream-colored isolates.

To date, the main methods used for determining phenolic compounds in plant raw materials include spectrophotometric techniques and high-performance liquid chromatography (HPLC)-based approaches. However, spectrophotometric methods can only determine the total content of phenolic compounds and have limited applicability due to the significant influence of complex matrix components, while HPLC requires expensive equipment, highly qualified personnel, long sample preparation, and destruction of samples during extraction of phenolic compounds (Aguilar-Hernández et al., 2017).

Raman spectroscopy and Fourier-transform infrared spectroscopy (FT-IR) are complementary techniques that provide fast, non-destructive, selective, yet simple real-time analysis. These methods allow for the analysis of objects in a variety of aggregate states. Additionally, vibrational techniques do not require complex sample preparation, which is one reason why they are often referred to as “green analytical techniques”. Raman and infrared spectroscopy (IR) are known as “fingerprint” methods because each molecule has a unique spectrum. Therefore, both methods are highly specific and suitable for label-free analysis. The identification and structure analysis of molecules are possible due to unique vibrational modes of certain functional groups in the analytes. At the same time, modern scientific literature contains single works devoted to applying vibrational spectroscopy methods to analyze phenolic compounds in plant materials. For instance, IR spectroscopy was used to determine the total phenolic compounds in apples (Pissard et al., 2013), tomatoes (Shan et al., 2014a), blueberries (Gajdoš Kljusurić et al., 2016) in reflectance mode, and in tea (Li et al., 2015) in transmission mode. Furthermore, FT-IR was used to determine polyphenols in extracts obtained from medicinal plant extracts (Wulandari et al., 2016), various fruits (Park et al., 2015), and potatoes (Shiroma-Kian et al., 2008). This method allows to detect phenolic compounds at levels of 0.05–15 wt% in food products (Lu and Rasco, 2012). Raman spectroscopy was employed for the determination of total phenols in carrots (Baranski et al., 2005) and pansy petals (Chafer et al., 2005). The study (Herdt et al., 2024) reveals the potential of Raman spectroscopy for tracking chlorogenic acid during roasting in coffee bean. In addition, Surface Enhanced Raman spectroscopy (SERS) has been used to determine phenolic compounds at lower concentrations (Maiti et al., 2013; Liu et al., 2022). Currently, most of the studies devoted to the use of vibrational spectroscopy techniques for quality control of plant materials describe procedures of extraction of target analytes from complex matrices. Such a sample preparation process requires the srlection of timing regimes and optimal reagent for efficient extraction.

In this manuscript we have combined the advantages of vibrational spectroscopies to develop highly efficient analysis of vegetable protein without prior extraction of phenolic compounds. In our work we developed a scalable non-destructive approach to control chlorogenic acid in protein-based matrices to obtain white to cream-colored isolates. Our detection approach is assessed by its required sensitivity for protein quality control and simplicity and in good agreement with HPLC analysis.

## 2 Materials and methods

### 2.1 Materials

The following reagents were used: potassium hydroxide KOH (“Helicon”, Russia), potassium bromide KBr (≥99%, “SigmaAldrich”, United States), bovine serum albumin (BSA) (≥98%, “Sigma-Aldrich”, United States), ethanol (technical), ferric chloride hexahydrate (“Merck”, Germany), potassium hexacyanoferrate (III) (“Merck”, Germany), Folin-Ciocalteu 2N reagent (“Sigma-Aldrich”, United States), hydrochloric acid (“Sigma Tech”, Russia), orthophosphoric acid (85%–90%, “Fulka”, Germany), acetonitrile (HPLC-grade, “Merck”, Germany).

The following analytes were also used: caffeic acid, C_9_H_8_O_4_ (≥98% “Acros organics”, Germany), D (−)-quinic acid C_7_H_12_O_6_ (≥98%, “Acros organics”, Germany), chlorogenic acid C_16_H_18_O_9_ (≥98%, “Acros organics”, India). Cold-pressed sunflower meal (produced by the Innovation Centre “Biryuch”) was used as a real object. Deionized water with a resistivity of at least 18.2 MΩ cm (“ULUPURE UPT-I-10T”, China) was used to prepare all the aqueous solutions.

### 2.2 Raman spectroscopy

Raman spectroscopy studies were performed using a confocal scanning Raman microscope “Horiba LabRAM HR Evolution” (HORIBA Ltd., Kyoto, Japan), equipped with a 514, 532, and 785 nm linearly polarized laser, diffraction grating with a 600 strokes/mm, and a ×50 objective lens. Unpolarized detection is used to provide a significant signal-to-noise ratio. Mapping tablets are formed using a pressing kit (9 mm) “PMY-B” (China).

#### 2.2.1 Registration of Raman spectra of phenolic acids standards

Powders of standard samples of chlorogenic acid, caffeic acid, and quinic acid were placed on a slide and Raman spectra were recorded using lasers with wavelengths of 514 (50 mW), 532 (50 mW), and 785 nm (90 mW). The signal accumulation time ranged from 3 s to 10 s, while the number of accumulations ranged from 1 to 10.

#### 2.2.2 Registration of Raman spectra of chlorogenic acid in BSA matrix and plotting the calibration curve

20 mg of chlorogenic acid standard was mixed with 180 mg BSA to obtain a model sample containing 10% chlorogenic acid in BSA. The samples were placed on slides, and Raman spectra were recorded using lasers with wavelengths of 514 nm, 532 nm, and 785 nm. The signal accumulation time was varied from 3 to 10 s, and the number of accumulations was varied from 1 to 10. The laser power was varied from 1% to 100% to select optimal conditions.

A series of mixtures with different concentrations of chlorogenic acid in a protein matrix was prepared by mixing and grinding 2, 4, 10, 14 and 20 mg of CGA with 198, 196, 190, 186 and 180 mg of BSA, respectively. The mixtures were then compacted into tablets using a pressure mold with a single-axis pressure of approximately 2 atm (∼200 kPa) for 1.5 min to form cylindrical tablets with a diameter of 9 mm. Additionally, similar tablets containing caffeic acid (CA) and quinic acid (QA) in BSA matrix were prepared. Mapping of the tablets was performed using laser scanning microscopy at a wavelength of 532 nm, on a 10 × 10 grid with a step size of 555 μm. The accumulation time was 10 s, and the number of accumulations was 2.

#### 2.2.3 Registration of Raman spectra of chlorogenic acid in sunflower meal matrix

20 mg of chlorogenic acid standard were mixed with 180 mg of sunflower meal (SFM) to obtain a model sample of chlorogenic acid in the SFM matrix (10 wt% chlorogenic acid in SFM). The follow-up procedure is similar to that described earlier for the BSA model.

### 2.3 Infrared spectroscopy

Infrared spectra have been recorded using a “Perkin Elmer Spectrum 3” FTIR spectrometer in transmission mode in the range 4,000–400 cm^−1^.

#### 2.3.1 Registration of IR spectra of phenolic acids standards

2 mg of chlorogenic acid standard were mixed with 148 mg of KBr, and then compacted into the form by pressing under the one-axis pressure on hydraulic press-machine (MTI Corporation YLJ-CSP-40A) of 2 atm (∼200 kPa) for 2 min. KBr was preliminary dehydrated at 300 °C for 4 h, using a muffle furnace (MTI Corporation KSL-1200X-UL). This process was repeated for caffeic and quinic acids using the same procedure.

#### 2.3.2 Registration of IR spectra of chlorogenic acid in BSA and SFM matrices and plotting the calibration curves

A series of 1, 2, 4, 10 and 20 mg of chlorogenic acid were mixed and ground with 199, 198, 196, 190, 180 mg of BSA, respectively, to obtain mixtures of 0.5%, 1%, 2%, 5% and 10% of CGA in the protein matrix. Similarly, 1, 2, 4, 6, 8, 14 and 20 mg of chlorogenic acid were mixed and ground with 199, 198, 196, 194, 186 and 180 mg of sunflower meal to obtain model mixtures of 0.5%, 1%, 2%, 3%, 4%, 7% and 10% of chlorogenic acid in the SFM matrix. 2 mg of each model sample were mixed and ground with 148 mg KBr and tablets were formed by pressing. IR spectra were obtained and calibration curves were plotted.

### 2.4 Spectrophotometric analysis

#### 2.4.1 Extraction of total phenolic compounds from sunflower meal

Total phenolic compounds from sunflower meal were extracted using water, 20%, 50% and 80% ethanol for 5, 15 and 30 min at a ratio of 1:20 weight to volume. The mixtures were centrifuged at 12,000 RPM for 10 min, and the resulting supernatants was collected.

#### 2.4.2 Determination of total phenolic compounds using the Folin-Ciocalteu method

To plot the calibration curve, 60 μL of chlorogenic acid standard solution (concentration range from 10 to 50 μg/mL), 60 μL of a two-fold Folin-Ciocalteu reagent and 60 µL of deionized water were successively added to a well of the 96-well plate. After 15 min, 120 μL of 10% sodium carbonate solution was added to the mixture. Then the mixture was left at room temperature (RT) in the dark for 1 hour to complete the reaction. Absorption spectra of the solutions were obtained at 760 nm using a microplate reader. 60 μL of a 100-fold diluted SFM extract, 60 μL of a two-fold Folin-Ciocalteu reagent, and 60 µL of deionized water were successively added to a well of the 96-well plate. Further, the analysis process is similar to that described above.

#### 2.4.3 Determination of total phenolic compounds using the prussian blue method

To plot the calibration curve, 100 μL of chlorogenic acid standard solution (concentration range from 10 to 50 μg/mL), 100 μL of 100 µL of 0.4 mM ferric (III) chloride solution and 100 µL of 0.36 mM potassium hexacyanoferrate (III) solution added to a well of the 96-well plate. Then the mixture was left at RT for 25 min to complete the reaction. Absorption spectra of the solutions were obtained at 700 nm using a microplate reader. 100 μL of a 100-fold diluted SFM extract, 100 μL of 100 µL of 0.4 mM ferric (III) chloride solution and 100 µL of 0.36 mM potassium hexacyanoferrate (III) solution added to a well of the 96-well plate. Further, the analysis process is similar to that described above.

### 2.5 HPLC

#### 2.5.1 Chromatographic conditions

An “Agilent 1,200” HPLC system with a spectrophotometric detector (wavelength of 326 nm and spectral bandwidth of 4 nm) was used. It was equipped with a “Synergi Hydro-RP” column (“Phenomenex”, United States), 150 × 4.6 mm, with a sorbent particle diameter of 4 μm. The temperature was kept at 30°C. Mobile phase A was 0.1% orthophosphoric acid, while mobile phase B was 80/20 vol% acetonitrile. The injection volume was 20 µL and the flow rate was 1 mL/min.

#### 2.5.2 Preparation of the mobile phase

0.1% of orthophosphoric acid was mixed with acetonitrile in the 80:20 volume ratio.

#### 2.5.3 Preparation of standard and calibration solutions of chlorogenic acid

1.0 mg of a chlorogenic acid standard solution was placed in a 100.0 mL volumetric flask. It was dissolved in 20.0 mL of mobile phase, then the volume of the solution was brought to the mark with mobile phase and stirred to ensure homogeneity. The concentration of the chlorogenic acid solution was 10 μg/mL 0.5; 1; 2.5; 5; 7.5 and 10 mL of chlorogenic acid standard solution were added to 10 mL volumetric flasks. The volume of each solution was adjusted to the mark using mobile phase, and then mixed. The concentrations of the calibration solutions ranged from 0.5 to 10 μg/mL of chlorogenic acid.

#### 2.5.4 Preparation of the test solution

0.5 mL of SFM extract obtained in step 2.4.1 is added to a 100 mL volumetric flask. The volume of the solutions is brought to the mark using mobile phase and then mixed.

#### 2.5.5 Chromatographic determination of chlorogenic acid

The mobile phase, five calibration solutions, and four test solutions were chromatographed in sequence. Each solution was analyzed three times.

### 2.6 Data analysis

The “Excel 2019” software was used to process the obtained data, Raman, IR- and UV-spectra were processed using “OriginPro 2018”. Background intensity subtraction was followed by integration of characteristic bands, and the area of these bands was chosen as an analytical signal. The chromatograms were also processed using the “OriginPro 2018”. For a standard solution of chlorogenic acid, the average peak area was calculated and the peak area was plotted as a function of acid concentration. Three determinations were carried out for the test solution, and the mean value of the content was calculated.

## 3 Results and discussion

### 3.1 Raman spectroscopy

#### 3.1.1 Modeling of Raman spectra of phenolic acids

**FIGURE 1:**
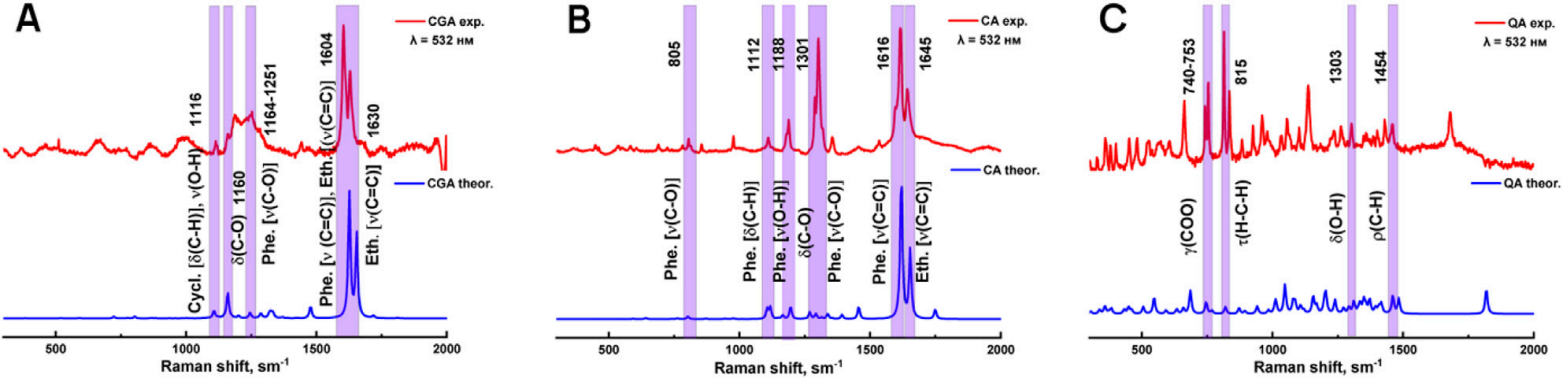
Modeled and experimentally obtained Raman spectra of chlorogenic **(A)**, caffeic **(B)** and quinic **(C)** acids.

Due to the lack of systematic literature study of Raman spectra of chlorogenic acid, caffeic acid and quinic acid, at the first stage experimentally obtained Raman spectra of individual phenolic acids were compared with model Raman spectra predicted by the DFT (density functional theory) method with a scaling factor of 0.9778 (Figure 1). Based on the calculations, the types of vibration of atoms in compounds in experimental Raman spectra were identified. The characteristic bands of chlorogenic acid, caffeic acid and quinic acid are located in the region between 700 and 1,700 cm^−1^, as shown in Table 1.

**TABLE 1:** Comparison of experimental and calculated Raman shifts of chlorogenic, caffeic and quinic acids.

Raman shift, cm^−1^ Theoretical data	Raman shift, cm^−1^ Experimental data	Vibrations
Chlorogenic acid		
1,107	1,116	ν(O-H), Phe. δ(ring), Phe. δ(C-H), Cycl. δ(ring)
1,160	1,160	δ(C-O), Eth. δ(C-H), δ(C-H)
1,197	1,197	Phe. δ(C-H), Phe. δ(O-H)
1,246	1,246	Phe. δ(C-H), Eth. δ(C-H), Phe. ν(C-O)
1,627	1,604	Phe. ν(С = С), Phe. δ(O-H), Eth. ν(C=C)
1,655	1,630	Eth. ν(C=C), Phe. ν(C=C), ν(C=O) ether
Caffeic acid		
804	805	Phe. δ(ring), Phe. ν(C-O)
975	975	Phe. δ(ring),, Phe. δ(C-H), Eth. δ(C-H)
1,109	1,109	Phe. δ(ring), Phe. δ(O-H), Eth. δ(C-H)
1,116	1,112	Phe. δ(ring), ν(C-O) in –COOH, Eth. δ(C-H)
1,196	1,188	Phe. ν(O-H), Phe. δ(C-H), Eth. δ(C-H)
1,268	1,268	δ(O-H) in –COOH, Phe. δ(C-H), Phe. ρ(C-H)
1,294	1,301	Phe. ν(C-O), Phe. δ(C-H)
1,619	1,616	Phe. ν(C=C), Eth. ν(C=C)
1,655	1,645	Eth. ν(C=C), Phe. ν(C=C)
Quinic acid		
685	685	δ(СOO)
748	748	δ(СOO)
822	815	τ(CH_2_)
1,044	1,055	ν(C-O), δ(O-H)
1,136	1,158	ν(C-O), δ(O-H)
1,240	1,240	δ(O-H), δ(С-H), ρ(СН_2_)
1,310	1,303	δ(O-H), δ(С-H), ρ(СН_2_)
1,460	1,454	ρ(СН_2_)
2,960	2,932	ν(C-H), δ(СН_2_)
3,004	2,975	ν(C-H), δ(СН_2_)

*Types of oscillations: ν–valence vibration; δ–strain scissor vibration; ω–strain fan vibration; τ–strain pendulum vibration, ρ - strain torsion vibration.

**Abbreviations: Phe. – phenyl nucleus; Eth. – ethylene; Cycl. – cyclohexane group.

The obtained data show that the set of characteristic modes in the theoretical and experimental spectra predominantly coincide, but their position and relative intensity for a number of bands differ. The peaks highlighted in purple were used for further analysis because they had the highest similarity between the theoretical and experimental values, and also because they were the most intense peaks.

To select the optimal conditions for obtaining Raman spectra, a standard sample of chlorogenic acid was examined using different lasers (514 nm, 532 nm, and 785 nm), varying the laser power (1%–100%) and signal accumulation time (3–10 s). The optimal conditions for recording the Raman spectrum have been chosen based on the visible vibration bands relative background at minimal laser power, time of measurement and number of accumulations to adopt this analysis for the real applications in future. Thus, conditions for measurement of chlorogenic acid are a laser with a wavelength of 532 nm, a power of 0.5 mW, an accumulation time of 10 s.

#### 3.1.2 Detection of chlorogenic acid in BSA matrix

**FIGURE 2:**
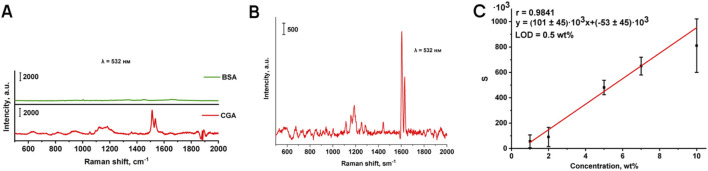
Comparison of the Raman spectra of BSA and chlorogenic acid to reveal mutual interfering influence **(A)**; Raman spectrum of chlorogenic acid in BSA matrix, 10 wt% **(B)**; Calibration curve for the determination of chlorogenic acid in BSA matrix **(C)**.

Further, the Raman spectra of a standard protein sample (BSA) and chlorogenic acid have been compared (Figure 2A), in order to determine the potential for identifying chlorogenic acid as dominant additive in real samples and to evaluate any interfering characteristic lines in the matrix. In the spectra of chlorogenic acid, there are closely spaced lines at 1,603 and 1,632 cm^−1^, corresponding to the valence vibrations of the C=C bond in the catechol nucleus and the ethylene group. Chlorogenic acid also has a band at 1,160 cm^−1^ (deformation vibration of the (C-O bond) in the ester group. Using a 532 nm laser of any power, BSA exhibits low Raman activity compared to chlorogenic acid and does not interfere with the detection of the characteristic bands of the analyte.

The Raman spectrum of chlorogenic acid in BSA matrix (10 wt% content) was obtained using a laser with a wavelength of 532 nm, and optimal conditions for its recording were selected (Figure 2B). When the studied model mixture is exposed to electromagnetic radiation at a wavelength of 532 nm, distinct characteristic bands are observed in the Raman spectrum of the chlorogenic acid on the background of the BSA. A smooth surface of the analyzed sample and a homogeneous distribution of chlorogenic acid within the protein were achieved by molding tablets with different weight fraction of chlorogenic acid in BSA. To construct the calibration curve, Raman mapping of pellets was performed with a step size of 550 µm using a λ = 532 nm laser. Figure 2C shows the calibration curve of the peak area of 1,605 cm^−1^ (S) versus the concentration for the determination of chlorogenic acid in the range from 1 to 10 wt% in BSA matrix, with a correlation coefficient of 0.9841.

The wide confidence intervals and high relative standard deviation (Table 2) are due to the peculiarities of the analysis method used. The diameter of the green laser beam is about 2 microns, using a lens with 50 times magnification. Consequently, the probability of focusing the beam on the analyte, which is distributed in a relatively small amount (1–10 wt%), in the BSA tablet, is rather low. That is why we used an automated approach based on accumulating a large number of Raman spectra with spatial resolution at relatively large increments, thereby creating the possibility of obtaining statistical data (100 points per tablet).

**TABLE 2:** Metrological characteristics of the technique for determining chlorogenic acid in BSA matrix using Raman spectroscopy.

The substance to be determined	The equation of the calibration curve	The range of determinable content, wt%	LOD, wt%	Correlation factor, R	S_r_ (n = 5 P = 0.95)
Chlorogenic acid	I = (100.56 ± 45.41) × 10^3^ C + + (−53.06 ± 45.41) × 10^3^	1–10	0.5	0.9841	0.84

Thus, Raman spectroscopy is an express multiplex approach that is potentially applicable to the analysis of plant proteins and isolates for chlorogenic acid with LOD of 0.5 wt%. In the future, it will be possible to achieve high sensitivity as well as a low detection limit using a modern and rapidly developing vibrational method, SERS.

### 3.2 Infrared spectroscopy

#### 3.2.1 Modeling of infrared spectra of phenolic acids

**FIGURE 3:**
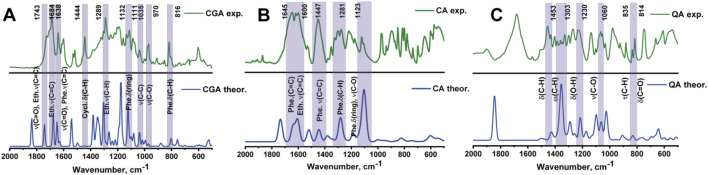
Theoretical and experimental IR spectra of chlorogenic **(A)**, caffeic **(B)** and quinic **(C)** acids.

Figure 3 shows the theoretical and experimental infrared (IR) spectra of chlorogenic acid, caffeic acid and quinic acid in the mid-infrared region (2,000–500 cm^−1^). The spectra were calculated using density functional theory (DFT) with a scaling factor of 1.0000. Overall, the model spectra agree well with the experimental data, with some differences in relative intensities. The characteristic bands and their corresponding vibrations are described in Table 3. The peaks highlighted in purple were used for further analysis because they had the highest similarity between the theoretical and experimental values, and also because they were the most intense peaks.

**TABLE 3:** Comparison of theoretical and experimental variations of chlorogenic, cinnamic and caffeic acids.

Wavenumber, cm^−1^ theoretical	Wavenumber, cm^−1^ Experimental	Vibrations
Chlorogenic acid		
802	816	Phe. δ(C-H), Cycl. ν(C-C), δ(O-H) в COOH
972	970	ν(C-O) в COOH, Phe. ν(C-O), ν(C-H), Phe. ω(C-H), Phe. δ(ring), Cycl. δ(ring)
1,035	1,035	ν(C-C), Cycl. δ(ring)
1,119	1,111	Phe. δ(ring), Phe. ν(C-O), Phe. ρ (C-H)
1,304	1,289	Eth. ν(С-H), Phe. ν(C-C), Phe. δ(C-H)
1,444	1,444	Cycl. δ(C-H)
1,648	1,638	Phe. ν(C-C), Phe. ν(C=C), Eth. ν(C=C), ν(C=O) ether
1,679	1,684	Phe. ν(C-C), Phe. ν(C=C), Eth. ν(C=C), Eth. δ(C-H), ν(C=O) ether
1743	1743	Eth. ν(C=C), ν(C=O) ether, Eth. δ(C-H)
Caffeic acid		
1,129	1,123	Phe. δ(riνg), Phe. ν(C-O)
1,310	1,310	Phe. δ(C-H), Phe. ν(C-O)
1,410	1,450	Phe. ν(C=C), δ(C-C), Phe. δ(O-H)
1,475	1,450	Phe. ν(C=C), Phe. ν(C-C), δ(C-C)
1,643	1,600	Phe. ν(C=C), Phe. ν(C-C), Eth. ν(C=C), ν(C-C), Phe. δ(C-H)
1,675	1,645	Phe. ν(C=C), Phe. ν(C-C), Eth. ν(C=C), ν(C-C), Phe. δ(C-H)
Quinic acid		
832	814	ν(С-О) in –COOH, δ(C=O), δ(C-H) in –COOH
1,025	1,029	ν(С-С)
1,060	1,060	ν(С-О)
1,100	1,100	ν(C-O)
1,215	1,230	δ(O-H), ρ (СН_2_)
1,354	1,354	ρ (СН_2_)

*Types of oscillations: ν–valence oscillation; δ–strain scissor oscillation; ω–strain fan oscillation; τ–strain pendulum oscillation, ρ - strain torsion oscillation.

**Abbreviations: Phe. – phenyl nucleus; Eth. – ethylene; Cycl. – cyclohexane group.

#### 3.2.2 Determination of chlorogenic acid in model mixtures with BSA

The ability to determine chlorogenic acid using IR spectroscopy depends on whether its characteristic bands can be observed and whether they do not overlap with the bands of the protein matrix. Since chlorogenic acid is a complex ester of caffeic and cinnamic acids, a band corresponding to the vibration of the ester group (-COO) at 1743 cm^−1^ should be present in the spectrum of chlorogenic acid. However, the lines in the region of 1,630–1750 cm^−1^ are very close together, resulting in poor peak resolution. Therefore, the peak with wavenumber at 1,444 cm^−1^ was chosen as the characteristic band corresponding to strain scissor oscillation of the cyclohexane group.

**FIGURE 4:**
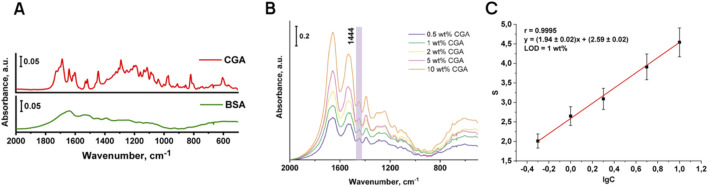
Comparison of IR spectra of BSA and chlorogenic acid **(A)**; IR spectra of model mixtures of chlorogenic acid in BSA matrix **(B)** and the corresponding calibration curve **(C)**.

As can be seen in Figure 4A, the characteristic bands of BSA are not located in the fingerprint region, so the model protein does not interfere with the determination of chlorogenic acid. Figure 4B illustrates the IR spectra of chlorogenic acid in the BSA matrix. To plot the calibration curve, the integral absorbance value (S) for the peak at 1,444 cm^−1^ and the decimal logarithm of the chlorogenic acid concentration in the model mixture were calculated (Figure 4C).

Analytical characteristics of the signal are given in Table 4. The correlation factor for the obtained calibration curve was 0.9995, which confirms good linearity of the described method for the determination of chlorogenic acid. The LOD of chlorogenic acid is 1 wt%. At the same time, in KBr tablets the LOD of the studied phenolic acid is exceptionally low for IR spectroscopy, reaching 6.7 × 10^−3^–1.3 × 10^−2^ wt% (bearing in mind that the analysis of the phenolic acid was carried out in the BSA matrix) and far ahead of previously presented IR-sensitivity for phenolic compounds in plant matrix. Thus, in paper (Shan et al., 2014a), chlorogenic acid was determined in coffee beans using NIR in diffuse reflectance mode at (4.78 ± 2.06) %.

**TABLE 4:** Metrological characteristics of the technique for determining chlorogenic acid in BSA matrix using IR spectroscopy.

The substance to be determined	The equation of the calibration curve	The range of determinable content, wt%	LOD, wt%	Correlation factor, R	RSD* (n = 5, P = 0.95)
Chlorogenic acid	A = (1.94 ± 0.02) × C + (2.59 ± 0.02)	1–10	1	0.9995	18

*Relative Standard Deviation (RSD).

#### 3.2.3 Determination of chlorogenic acid in sunflower meal by additive method

**FIGURE 5:**
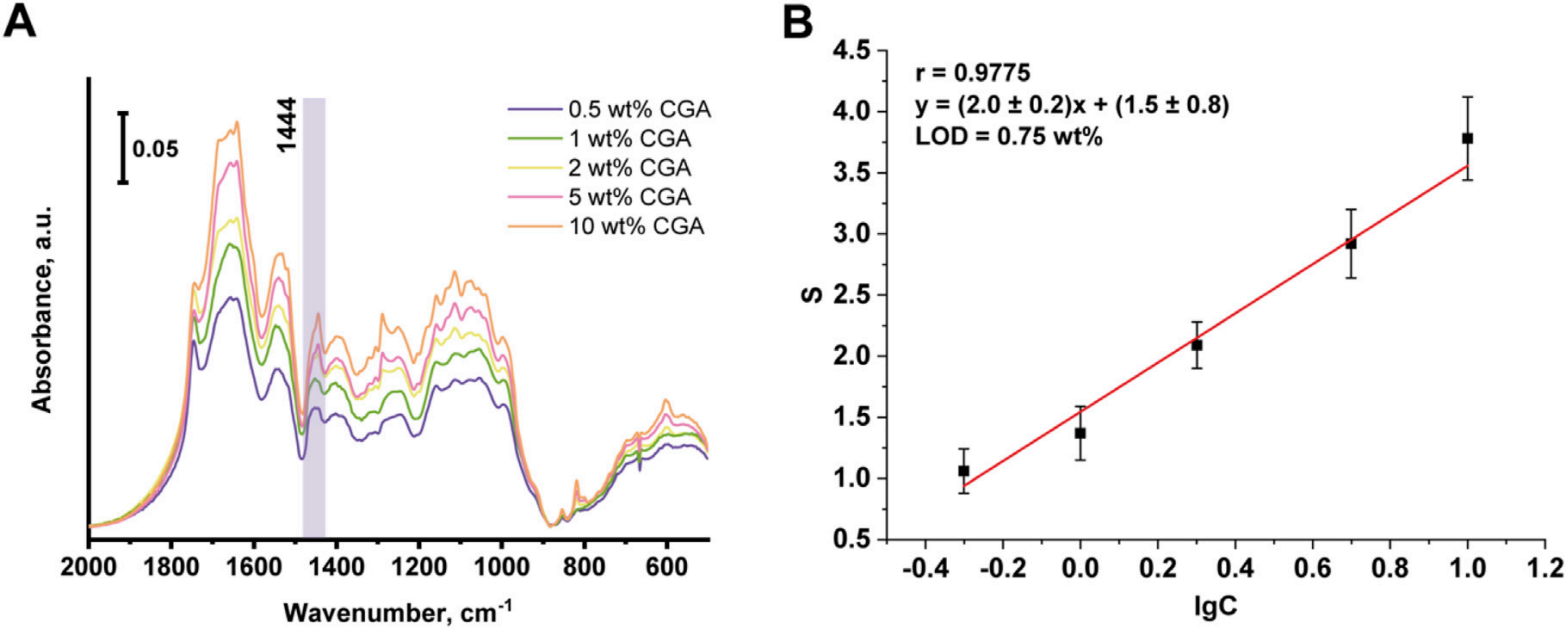
IR spectra of SFM with addition of chlorogenic acid **(A)** and the corresponding calibration curve **(B)**.

When IR spectra of model mixtures of chlorogenic acid in sunflower meal matrix were recorded, a characteristic peak at the area of 1,444 cm^−1^ was also observed. Figure 5 shows the IR spectra of sunflower meal with added chlorogenic acid content ranging from 0.5% to 10%, and the corresponding calibration curve of the integral absorbance value (S) for the peak at 1,444 cm^−1^ versus decimal logarithm of the chlorogenic acid concentration in the model mixture. Metrological characteristics are shown in Table 5. The research found that the matrix of the sunflower meal did not interfere with the determination of the chlorogenic acid. The correlation factor for the obtained calibration curve was 0.9775, the LOD of chlorogenic acid is 0.75 wt%. Therefore, the content of chlorogenic acid in sunflower meal, determined by the additive method, was (5.6 ± 0.8) wt%.

**TABLE 5:** Metrological characteristics of the technique for determining chlorogenic acid in SFM using IR spectroscopy.

The substance to be determined	The equation of the calibration curve	The range of determinable content, wt%	LOD, wt%	Correlation factor, R	RSD (n = 5,P = 0.95)
Chlorogenic acid	А = (2.0 ± 0.2) × C + (1.5 ± 0.8)	0.5–10	0.75	0.9775	12

### 3.3 Spectrophotometric analysis

**FIGURE 6:**
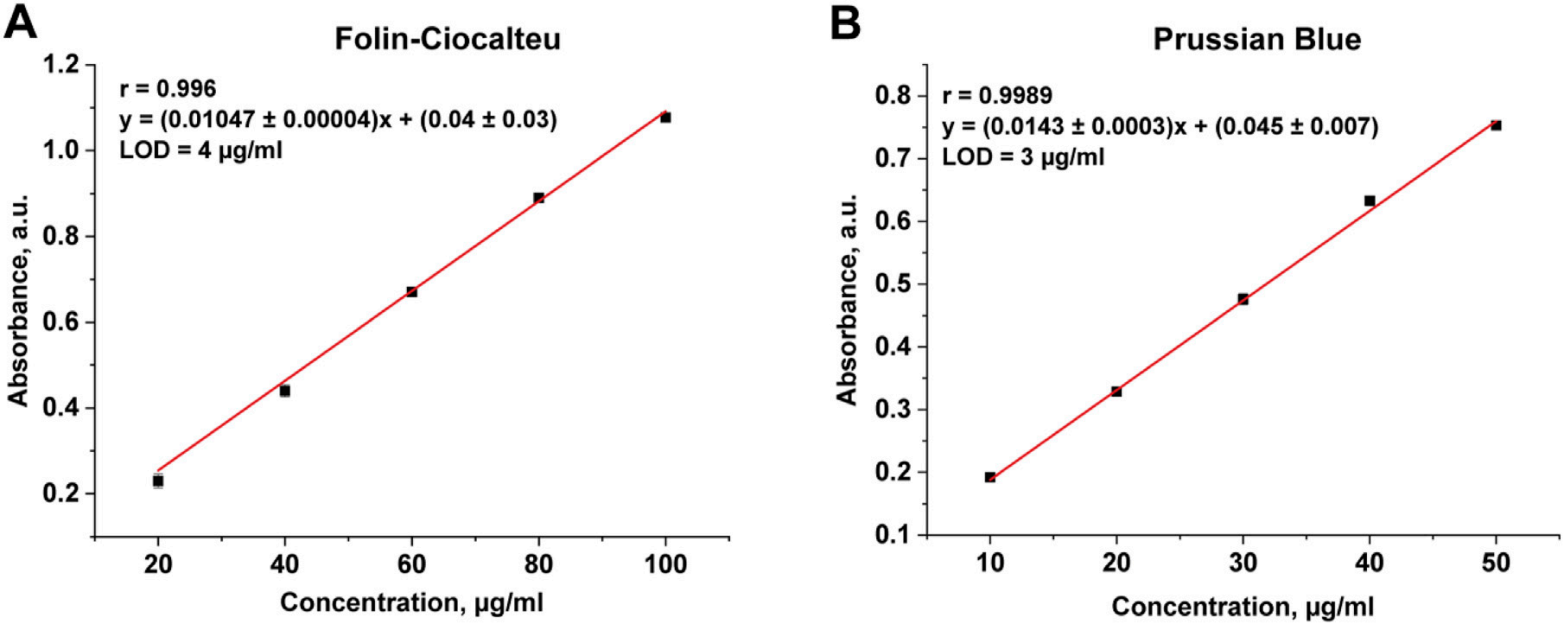
The calibration curve for Folin-Ciocalteu method **(A)**, Prussian Blue method **(B)**.

Currently, it is known about the application of spectrophotometric methods for the determination of the total content of phenolic compounds in plant materials. For example, in manuscript (Margraf et al., 2015) both described methods (Folin-Ciocalteu and Prussian Blue) were used for the determination of phenolic compounds in tea and grape juices. In addition, the Folin-Ciocalteu method was used for the determination of phenolic compounds in Robusta coffee brews (Jeszka-Skowron et al., 2016) and plant tissue extracts (Nikolaeva et al., 2021), and the Prussian Blue method was used for the determination of chlorogenic acid in fermentation broth and mango (Wang et al., 2019). Therefore, we used spectrophotometric methods to verify the results of IR analysis. Since the determination of phenolic compounds by Folin-Ciocalteu and Prussian Blue spectrophotometric methods requires prior sample preparation and their extraction from the sunflower meal matrix, optimal extraction conditions were chosen at the beginning of the research. For this purpose, the effects of ethanol concentration and extraction time on the yield of phenolic compounds were compared. The calibration curves for both methods were plotted using chlorogenic acid as a standard (Figure 6). The range of determinable content for FC method was 20–100 μg/mL, for PB method – 10–50 μg/mL. The correlation factor for both methods was higher than 0.99, confirming the linearity.

As can be seen in Table 6, extraction using 50% ethanol during 30 min was the most efficient. Folin-Ciocalteu data showed the total phenolic content of sunflower meal varied between (2.74 ± 0.01) and (4.82 ± 0.01) %. Prussian blue data showed slightly higher levels from (5.22 ± 0.02) to (5.52 ± 0.02) %, that can be explained by the sensitivity of the reagents and the different redox potentials of the matrix. The determined content of phenolic compound is in good qualitative agreement with the value obtained by IR-spectroscopy. Notably, the application of these spectrophotometric methods for the determination of total phenolic compounds in sunflower meal was demonstrated for the first time.

**TABLE 6:** Results of extraction of phenolic compounds from sunflower meal.

Extraction conditions	Extraction time					
	5 min		15 min		30 min	
	FC	PB	FC	PB	FC	PB
	Total content of phenolic compounds, %					
80% EtOH	2.74 ± 0.01	3.51 ± 0.01	3.18 ± 0.03	3.67 ± 0.01	3.57 ± 0.01	4.43 ± 0.01
50% EtOH	4.07 ± 0.02	5.29 ± 0.01	4.18 ± 0.01	5.22 ± 0.02	4.82 ± 0.01	5.52 ± 0.02
20% EtOH	4.54 ± 0.01	4.76 ± 0.02	4.88 ± 0.02	5.16 ± 0.01	4.63 ± 0.01	5.13 ± 0.01
H2O	4.35 ± 0.01	4.45 ± 0.01	4.51 ± 0.01	4.66 ± 0.01	3.91 ± 0.02	5.26 ± 0.01

### 3.4 HPLC

HPLC is another widely used ultra-sensitive method for the determination of phenolic compounds. Importantly, it requires the destruction of the raw sample. In contrast to spectrophotometric methods based on the determination of the total content of phenolic compounds, HPLC allows the determination of individual substances. There are known single works devoted to the determination of chlorogenic acid in sunflower meal extracts. Thus, in research (Náthia-Neves and Alonso, 2021), the content of chlorogenic acid in a sample obtained after microwave extraction of sunflower meal was found at 8.4 ± 0.1 mg CGA/g of raw material. As chlorogenic acid is the major phenolic compound in sunflower meal, it was used as a standard. The HPLC profile of the SFM extracts corresponded to the chromatogram of a standard sample of chlorogenic acid, with retention times about 2.7 min (Table 7). The content of chlorogenic acid in the sample obtained by extraction under optimum conditions (50% ethanol, 30 min) was (3.62 ± 0.02) %, which is in general agreement with IR spectroscopy and spectrophotometry data.

**TABLE 7:** Results of determination of chlorogenic acid in sunflower meal by HPLC method with UV detection.

Extraction conditions	Retention time of CGA, min	Content in the sample, %
80% EtOH, 30 min	2.7	3.07 ± 0.03
50% EtOH, 30 min	2.7	3.62 ± 0.02
20% EtOH, 30 min	2.7	3.34 ± 0.02
H_2_O, 30 min	2.7	2.37 ± 0.01

Moreover, spectrophotometric and HPLC methods have lower confidence intervals than vibrational spectroscopy methods. However, these methods require preliminary sample preparation–extraction of phenolic compounds from the sunflower meal matrix compared to the vibrational techniques. At the same time, changing the conditions (solvent, time, temperature) during sample preparation may change the content of phenolic compounds in the extract with a value of deviation from each other of one order more than the confidence interval for each sample (Tables 6, 7). Vibrational spectroscopy methods do not have this disadvantage. It makes the analytical characteristics of the signal, determined by vibrational spectroscopy, similar to those obtained by spectrophotometric and HPLC methods, and proves the possibility of multiplex determination of the phenolic compounds at the level of reference concentrations in protein isolates as well as promising future facilitation of protein control using Raman and infrared spectroscopic analysis.

## 4 Conclusion

The manuscript proposes a new, non-destructive approach to the quality control of sunflower meal and its by-products. The principal possibility of analyzing protein isolates using Raman scattering has been demonstrated, with the LOD for chlorogenic acid content of 1 wt%. In order to determine lower concentrations of phenolic compounds, Surface Enhanced Raman spectroscopy can be used. IR spectroscopy is suitable for simple non-destructive analysis of raw sunflower meal for chlorogenic acid. The chlorogenic acid concentration of the sample was determined by this method to be (5.6 ± 0.8) %, which was confirmed by spectrophotometric and HPLC methods. On the basis of the study, it can also be concluded that if the matrix is inactive, but the phenolic compounds contained therein are Raman and/or IR active, it is possible to apply the described approach to other plant materials. Furthermore, the application of the mathematical modelling technique (DFT) can help to identify bioactive compounds in other kind of products.

## Data Availability

The original contributions presented in the study are included in the article/supplementary material, further inquiries can be directed to the corresponding author.
